# 4238 Racial/ethnic variation in the relation between diabetes control and healthy eating, food security, exercise, and access to health care

**DOI:** 10.1017/cts.2020.423

**Published:** 2020-07-29

**Authors:** Magda Shaheen, Quanincia Sanderlin, Katrina Schrode

**Affiliations:** 1 David Geffen School of Medicine at UCLA; 2 Charles R Drew University

## Abstract

OBJECTIVES/GOALS: Diabetes mellitus is a common metabolic disease. Uncontrolled diabetes can lead to complications. The objective of this study is to examine the racial/ethnic variation in the relation between diabetes control and healthy eating, food security, exercise, and access to health care. METHODS/STUDY POPULATION: We analyzed data related to diabetes control, demographics, insurance, healthy eating, food security and physical activity for 949 diabetics from the National Health and Nutrition Examination Survey (NHANES). The population examined was adults with diabetes mellitus. Diabetes control was classified as fair control [HbA1c 7-<8 and fasting glucose <126 mg/dL], good control [HbA1c<7 and fasting glucose <110 mg/dL] or uncontrolled [HbA1c8 and fasting glucose <110 mg/dL]. We used multinomial logistic regression controlling for confounders to analyze the data overall and for each racial/ethnic group and report adjusted odds ratios (AOR) and 95% confidence limits (CL). RESULTS/ANTICIPATED RESULTS: Of the 949 diabetics, 14.7% were Blacks, 15.9% were Hispanics, 11.0% had fair control, 61.0% had good control, 14.2% were uninsured, 18.1% had low/very low food security, and 39.7% were inactive. Overall, uninsured subjects had a lower chance of fair diabetes control (AOR = 0.2, 95% CL = 0.1-0.9, p = 0.04), but this relationship was significant only for Hispanics and Blacks (p<0.05). Whites with low/very low food security were less likely to have fair control (AOR = 0.2, 95% CI = 0.001-0.9, p = 0.04). Blacks who were inactive, and insufficiently active Hispanics had a lower chance of good/excellent diabetes control (AOR = 0.5, 95% CI = 0.2-0.9, p = 0.03; AOR = 0.3, 95% CI = 0.1-0.7, p = 0.007 respectively). DISCUSSION/SIGNIFICANCE OF IMPACT: The results show the importance of insurance coverage, food security and physical activity in diabetes control among different racial/ethnic groups. They indicate a need for affordable health care and for culturally-relevant interventions that include physical activity and food security.

